# Tandem repeat disorders

**DOI:** 10.1093/emph/eoz005

**Published:** 2019-01-28

**Authors:** Calen P Ryan

**Affiliations:** Department of Anthropology, Northwestern University, Evanston, IL, USA

## TANDEM REPEAT DISORDERS

**Figure 1.**
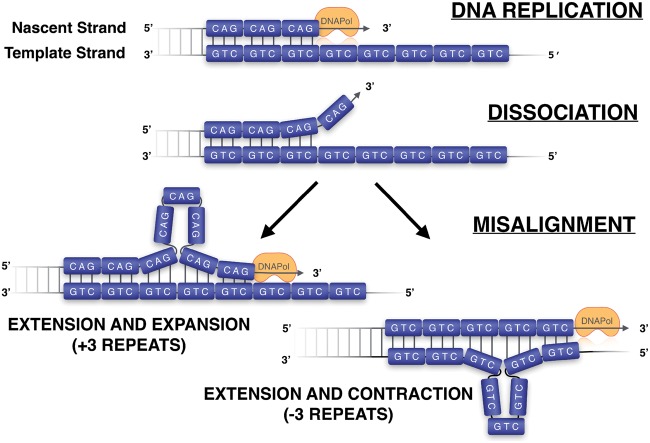
Replication slippage: DNA strands disassociate and misalign, leading to expansion/contraction in short-tandem repeat number, here a glutamine (CAG). DNAPol, DNA Polymerase

Tandem repeat disorders (TRDs) are a family of neuropathological disorders linked to the accumulation of short-tandem repeats (STRs; repeating DNA sequences 2–6 basepairs in length) (Fig. 1). TRDs arise with STR number expansion from normal to pathological, a number that varies by disorder. TRDs account for >20 heritable neuropathologies, including Huntington’s disease, Kennedy’s disease, myotonic dystrophy, Fragile X syndrome and several spinocerebellar ataxias [1].

STRs comprise ∼3% of the human genome. Repeat hyper-expansion within almost all genomic contexts, including in non-coding regions, can result in pathology. TRDs can arise from a disruption in the structure, function, and/or quantity of RNA, proteins and/or local epigenetic processes. A number of TRDs, including Huntington’s disease, occur in the context of expanded glutamine (CAG) repeats, accompanied by protein misfolding, aggregation, and the toxicity [2].

## EVOLUTIONARY PERSPECTIVES

STRs have the capacity to expand (or contract) through ‘replication slippage’ (Fig. 1). Replication slippage occurs when the template and nascent strands disassociate during DNA replication, misaligning and creating hairpin loops. These ‘loops’ can cause repeat contraction (if on the template strand) or expansion (on the nascent strand) (Fig. 1). More opportunities for misalignment make longer repeat tracts especially prone to slippage and expansion/contraction events and hence more mutable. Other factors, including repeat unit size and base pair composition, affect the mutability of repeats contributing to TRDs [2].

Despite numerous pathological TRDs, long and potentially unstable repeat tracts are common in the human genome [3, 4]. This characteristic may arise through conflict between protein and DNA. Natural selection should favor high-fidelity DNA replication and hence fewer STRs, while protein form and function may require structural features such as loops or helices that rely on tandemly repeated DNA [5]. However, natural variation in STRs is also associated with rapidly evolving morphological and behavioral traits (i.e. neural development and function in humans) [4]. The capacity for STRs to modulate incremental phenotypic effects over evolutionarily brief timescales has led to their proposed role as ‘evolutionary tuning knobs’ [2].

## CLINICAL RELEVANCE

The highly mutable nature of STRs contributes to the heritability and disease characteristics of TRDs. A *de novo* repeat expansion occurring in sperm/egg can result in a heritable disease phenotype within a single generation. Additional expansion events often worsen the severity of symptoms or shorten the age of onset with each generation, a process known as ‘anticipation’. The repetitive nature, dynamic evolution, and complex heritability of STRs have made their study *in vivo* challenging. However, improved algorithms for alignment of repetitive DNA and single-cell technologies promise to clarify the role of STRs in human health, evolution, and phenotypic variation.
